# Association Between Hypertensive Disorders of Pregnancy and Neurodevelopmental Outcomes Among Offspring

**DOI:** 10.1001/jamapediatrics.2020.6856

**Published:** 2021-03-22

**Authors:** Judith S. Brand, Deborah A. Lawlor, Henrik Larsson, Scott Montgomery

**Affiliations:** 1 Clinical Epidemiology and Biostatistics, School of Medical Sciences, Örebro University, Örebro, Sweden; 2 Medical Research Council Integrative Epidemiology Unit, University of Bristol, Bristol, United Kingdom; 3 Population Health Sciences, Bristol Medical School, Bristol, United Kingdom; 4 School of Medical Sciences, Örebro University, Örebro, Sweden; 5 Department of Medical Epidemiology and Biostatistics, Karolinska Institutet, Stockholm, Sweden; 6 Clinical Epidemiology Division, Department of Medicine, Solna, Karolinska Institutet, Stockholm, Sweden; 7 Department of Epidemiology and Public Health, University College London, London, United Kingdom

## Abstract

**Importance:**

Hypertensive disorders of pregnancy (HDP) have been associated with poorer neurodevelopmental outcomes in offspring, but the role of familial confounding in these associations is unclear.

**Objective:**

To investigate associations of maternal HDP with risks in offspring of autism spectrum disorders (ASDs), attention-deficit/hyperactivity disorder (ADHD), and intellectual disability (ID), as well as variation in overall cognitive performance in offspring.

**Design, Setting, and Participants:**

This Swedish register-based study used data from a birth cohort divided into 1 085 024 individuals born between 1987 and 1996 and followed up until December 31, 2014, and 285 901 men born between 1982 and 1992 who attended assessments for military conscription, including a cognitive function test. Statistical analysis was performed from April 1, 2019, to June 1, 2020.

**Exposures:**

Diagnoses of HDP, which were provided by the Medical Birth Register.

**Main Outcomes and Measures:**

Diagnoses of ASDs, ADHD, and ID were extracted from the National Patient Register. Cognitive function was assessed using written tests and summarized as a single 9-point score. Whole-cohort and within-sibship analyses were performed; the latter accounted for unmeasured familial confounding factors shared by siblings.

**Results:**

The study included 1 085 024 individuals (556 912 male participants [51.3%]) born between 1987 and 1996 and 285 901 men born between 1982 and 1992 who attended assessments for military conscription. The prevalence of maternal HDP was 4.0% in the 1987-1996 birth cohort (n = 42 980) and 5.1% in the military conscription cohort (n = 14 515). A total of 15 858 participants received a diagnosis of ASD, 36 852 received a diagnosis of ADHD, and 8454 received a diagnosis of ID. The mean (SD) cognitive score among the men in the conscription cohort was 5.1 (1.9). In whole-cohort analyses with multivariable adjustment, HDP were associated with offspring ASDs (hazard ratio [HR], 1.22; 95% CI, 1.13-1.31), ADHD (HR, 1.10; 95% CI, 1.05-1.16), and ID (HR, 1.39; 95% CI, 1.27-1.53). Analyses comparing siblings discordant for HDP were less statistically powered but indicated estimates of similar magnitude for ASDs (HR, 1.19; 95% CI, 1.00-1.42) and possibly ADHD (HR, 1.09; 95% CI, 0.95-1.24), but not for ID (HR, 1.04; 95% CI, 0.83-1.29). Hypertensive disorders of pregnancy were associated with somewhat lower cognitive scores in whole-cohort analysis (mean difference comparing offspring exposed with those unexposed, −0.10; 95% CI, −0.13 to −0.07), but in within-sibship analysis, the association was null (mean difference, 0.00; 95% CI, −0.09 to 0.08).

**Conclusions and Relevance:**

The study results suggest that HDP are associated with small increased risks of ASDs and possibly ADHD in offspring, whereas associations with ID and cognitive performance are likely confounded by shared familial (environmental or genetic) factors.

## Key Points

**Question:**

Are hypertensive disorders of pregnancy (HDP) associated with poorer neurodevelopmental outcomes in offspring independently of shared familial confounding factors?

**Findings:**

In this cohort study, offspring of HDP-complicated pregnancies had a somewhat higher incidence of autism spectrum disorders (ASDs), attention-deficit/hyperactivity disorder (ADHD), and intellectual disability (ID) and slightly lower overall cognitive performance. Analyses comparing siblings had less statistical power and indicated associations of a similar magnitude with ASDs and possibly ADHD only.

**Meaning:**

This study suggests that HDP are associated with modestly increased risks of ASDs and possibly ADHD in offspring, whereas associations with ID and cognitive performance are likely the result of confounding by shared familial characteristics.

## Teaser

This cohort study investigates associations of maternal hypertensive disorders of pregnancy with risks in offspring of autism spectrum disorders, attention-deficit/hyperactivity disorder, and intellectual disability, as well as variation in overall cognitive performance in offspring.

## Introduction

Hypertensive disorders of pregnancy (HDP), a group of disorders that encompasses both chronic hypertension and de novo hypertension in pregnancy, are common, affecting 5% to 10% of all pregnant women.^1,2^ Apart from being a major cause of perinatal mortality, fetal growth restriction, and preterm birth,^3^ accumulating evidence suggests that HDP may have long-term neurodevelopmental consequences for the offspring.^4^ Because placental perfusion is suboptimal in HDP-complicated pregnancies, reduced oxygen and nutritional supplies may be associated with alterations in fetal brain development and subsequent poorer behavioral and cognitive development.^5,6^

Several studies have reported an increased incidence of neurodevelopmental disorders, including autism spectrum disorders (ASDs) and attention-deficit/hyperactivity disorder (ADHD),^5,7,8,9,10^ among offspring born to mothers with HDP. In addition, some,^11,12^ but not all,^13,14^ studies have found a higher incidence of intellectual disability (ID) and lower cognitive performance scores among offspring of HDP-complicated pregnancies. However, evidence as to whether reported associations represent true causal intrauterine effects has been judged as being insufficient.^4,15^

Studies examining associations of prenatal exposures with offspring health outcomes are prone to residual confounding by environmental and genetic factors that are shared within families. Hypertensive disorders of pregnancy have been associated with sociodemographic and lifestyle characteristics,^16,17,18^ factors that tend to cluster within families and may determine the likelihood of offspring receiving a diagnosis of a neurodevelopmental outcome. Familial confounding by shared genetic factors may also exist because both traits are genetically determined, with recent evidence suggesting an association between maternal risk alleles for neurodevelopmental disorders (primarily ADHD) and pregnancy-related exposures.^19^ Because it would be impossible to randomize women with HDP, observational studies investigating the causal role of maternal HDP in offspring’s neurodevelopmental outcomes need to consider potential residual confounding by these shared familial factors.^20^

In this study, we examined the association of HDP with offspring ASDs, ADHD, and ID in a Swedish register-based birth cohort. In addition, we assessed the association with overall cognitive performance in male adolescents who were conscripted for military service in Sweden. To strengthen causal inference, we compared results from whole-cohort and within-sibship analyses, with the latter accounting for unmeasured familial confounding factors.

## Methods

### Study Population

We analyzed data from a birth cohort divided into 1 085 024 individuals born between 1987 and 1996 and followed up until December 31, 2014 (hereafter referred to as the “1987-1996 birth cohort”), and 285 901 men born between 1982 and 1992 who attended assessments for conscription into the Swedish military service at around 18 years of age (referred to as the “military conscription cohort”). These 2 subcohorts were assembled by merging data from various Swedish registers (eMethods and eFigure in the Supplement). Siblings in these cohorts were identified using the Multi-Generation Register,^21^ which enables identification of first-degree relatives of each index individual. Permission for the study was granted by the Swedish Ethical Review Agency. Because this is a register-based study, informed consent was waived.

### Hypertensive Disorders of Pregnancy

Maternal HDP diagnoses were identified in the Medical Birth Register^22^ using *International Classification of Diseases, Eighth Revision* (*ICD-8*) codes 401 and 637 and *International Classification of Diseases, Ninth Revision* (*ICD-9*) code 642.^10^ These codes do not differentiate between specific HDP diagnoses but combine chronic hypertension (predating pregnancy or diagnosed before 20 weeks of gestation), gestational hypertension, and preeclampsia.^23^ Diagnostic criteria for gestational hypertension and preeclampsia specify that there is elevated blood pressure (in those without chronic hypertension) after 20 weeks of gestation. Because chronic hypertension is relatively rare in women of reproductive age, most HDP diagnoses will identify gestational hypertension or preeclampsia.

### Outcomes

#### Neurodevelopmental Disorders

We used the 1987-1996 birth cohort to examine associations with ASDs, ADHD, and ID among offspring, identified as any first outpatient or inpatient main diagnosis in the National Patient Register,^24^ based on relevant *International Classification of Diseases* codes (eTable 1 in the Supplement). Previous research suggests that risk factors for ASDs may differ depending on comorbid ID.^25,26^ We therefore also examined associations with ASDs in the presence or absence of ID separately.

#### Overall Cognitive Performance

The association with overall cognitive performance was examined in the military conscription cohort, consisting of men who underwent a full medical assessment before entering military service, including written cognitive function tests covering logical, spatial, verbal, and technical abilities. The results of these tests were transformed into a single score and normalized on a standard scale ranging from 1 to 9, where 1 indicates lowest cognitive function and 9 indicates highest cognitive function (eMethods in the Supplement).

### Covariates

Information on calendar year of birth, sex, gestational age, and birth weight was obtained from the Medical Birth Register. Maternal characteristics (age at birth, parity, and height, body mass index [BMI], and smoking at the first antenatal visit) and diagnoses of pregestational diabetes (eTable 1 in the Supplement) were also extracted from this register. Linkage to the Multi-Generation Register^21^ combined with the LISA (Longitudinell Integrationsdatabas för Sjukförsäkrings–och Arbetsmarknadsstudier) database^27^ provided details on parental marital status, highest completed educational level, and occupational classification using an approximation of the European Socio-economic Classification^28^ around the time of birth of each offspring.

### Statistical Analysis

Statistical analysis was performed from April 1, 2019, to June 1, 2020. Associations with neurodevelopmental disorders were analyzed using Cox proportional hazards regression with follow-up time defined from birth until the date of diagnosis, emigration, death, or end of follow-up (December 31, 2014), whichever came first. The proportional hazards assumption was assessed graphically using scaled Schoenfeld residuals plots, showing no evidence of nonproportional hazards. Linear regression was used for examining associations with cognitive function scores, but we also evaluated the existence of a potential nonlinear association using multinomial logistic regression, including the scores as an ordinal outcome (low [1-3], medium [4-6], and high [7-9]).

Within-sibship analyses were conducted in a multilevel framework (siblings within families) by conditioning the analysis on the family identifier. The fixed-effects regression coefficients from these models provide estimates of the within-sibship association. We fitted 2 models for the whole-cohort and within-sibship analyses: (1) a model adjusted for year of birth only and (2) a multivariable-adjusted model including year of birth, maternal age, parity, height, and BMI; pregestational diabetes; parental educational level, occupation, and marital status; and offspring sex. The generalizability of the within-sibship results was assessed by repeating whole-cohort analyses for offspring with or without siblings. To investigate whether associations were mediated by timing of delivery and fetal growth, we performed model 2 with additional adjustment for gestational age at birth and birth weight for gestational age. All effect estimates are presented with 95% CIs. A 2-sided *P* < .05 was considered statistically significant.

In total, 507 479 offspring (46.8%) in the 1987-1996 birth cohort and 131 861 offspring (46.1%) in the military conscription cohort had missing data for 1 or more covariates. The level of missing data was highest for maternal BMI and height because these variables were not routinely recorded in the Medical Birth Register until 1992. Because missing values were likely to be missing at random and to avoid loss in statistical efficiency, missing values were imputed using multiple imputation (eMethods in the Supplement).

We further conducted several sensitivity analyses: (1) analyses with more refined definitions of ASDs, ADHD, and ID requiring at least 2 diagnoses more than 6 months apart; (2) analyses with additional adjustment for conditions other than HDP complicating pregnancy, birth, or the puerperium in offspring with this diagnostic information (eTable 1 in the Supplement); (3) analyses stratified by calendar period of birth to evaluate sensitivity to changing diagnostic criteria; (4) within-sibship analyses including full siblings only and analyses stratified by the between-sibling age difference within a family (≤3 and >3 years) to explore sensitivity to the degree of control for familial confounding; (5) within-sibship analyses by birth order of HDP occurrence; and (6) complete case analyses as alternative to multiple imputation.

## Results

The prevalence of maternal HDP was 4.0% in the 1987-1996 birth cohort (n = 42 980) and 5.1% in the military conscription cohort (n = 14 515). The Table summarizes the distribution of parental and offspring characteristics by presence of HDP. In both cohorts, mothers with HDP were more likely to be of older age (≥35 years), to be overweight or obese, and to have pregestational diabetes and other conditions complicating pregnancy, birth, or the puerperium. They were also more often nulliparous and less frequently smoked in early pregnancy. Offspring of mothers with HDP, on average, had parents with a lower educational level and routine or manual occupations, and they had a lower gestational age and weight at birth.

**Table.** Parental and Offspring Characteristics by Maternal HDP Status, Stratified by Cohort

Characteristic	No. (%)^a^			
	1987-1996 Birth cohort (n = 1 085 024)		Military conscription cohort (n = 285 901)	
	No HDP (1 042 044 [96.0])	HDP (42 980 [4.0])	No HDP (271 386 [94.9])	HDP (14 515 [5.1])
Maternal age, y				
<20	27 160 (2.6)	1276 (3.0)	7856 (2.9)	519 (3.6)
20-24	224 933 (21.6)	9943 (23.1)	62 289 (23.0)	3575 (24.6)
25-29	391 183 (37.5)	14 723 (34.3)	102 161 (37.6)	4964 (34.2)
30-34	274 169 (26.3)	10 439 (24.3)	69 226 (25.5)	3453 (23.8)
≥35	124 599 (12.0)	6599 (15.4)	29 854 (11.0)	2004 (13.8)
Mean (SD)	28.3 (5.0)	28.5 (5.5)	28.0 (5.0)	28.1 (5.5)
Maternal height, cm				
<155	24 707 (3.0)	973 (2.8)	5371 (2.7)	273 (2.5)
155-164	297 326 (36.2)	12 701 (36.8)	74 083 (36.7)	4102 (37.5)
165-174	429 557 (52.4)	17 973 (52.0)	106 947 (53.0)	5744 (52.6)
≥175	68 735 (8.4)	2891 (8.4)	15 269 (7.6)	808 (7.4)
Mean (SD)	166.1 (6.1)	166.1 (6.1)	166.1 (6.0)	166.0 (6.0)
Missing	221 719 (21.3)	8442 (19.6)	69 716 (25.7)	3588 (24.7)
Maternal BMI				
<18.5	31 018 (5.0)	767 (2.9)	13 048 (7.8)	400 (4.3)
18.5-24.9	448 425 (71.9)	15 484 (58.3)	131 117 (78.8)	6425 (69.2)
25.0-29.9	112 412 (18.0)	7062 (26.6)	19 028 (11.4)	1961 (21.1)
≥30.0	31 860 (5.1)	3268 (12.3)	3135 (1.9)	497 (5.4)
Mean (SD)	23.1 (3.6)	24.7 (4.5)	21.9 (3.0)	23.3 (3.6)
Missing	418 329 (40.1)	16 399 (38.2)	105 058 (38.7)	5232 (36.0)
Maternal parity				
Nulliparous	421 601 (40.5)	25 123 (58.5)	111 430 (41.1)	8113 (55.9)
1	380 155 (36.5)	10 355 (24.1)	99 041 (36.5)	3743 (25.8)
2	167 406 (16.1)	4940 (11.5)	44 884 (16.5)	1837 (12.7)
≥3	72 882 (7.0)	2562 (6.0)	16 031 (5.9)	822 (5.7)
Maternal smoking in early pregnancy				
No	756 680 (77.1)	34 120 (84.2)	175 207 (73.0)	10 190 (79.4)
1-10 Cigarettes/d	140 402 (14.3)	4132 (10.2)	40 471 (16.9)	1690 (13.2)
>10 Cigarettes/d	84 454 (8.6)	2279 (5.6)	24 360 (10.1)	956 (7.4)
Missing	60 508 (5.8)	2449 (5.7)	31 348 (11.6)	1679 (11.6)
Maternal pregestational diabetes	500 (0.0)	88 (0.2)	1097 (0.4)	131 (0.9)
Other conditions complicating pregnancy, birth, or the puerperium	42 984 (4.1)	3207 (7.5)	4398 (3.8)	287 (6.0)
Missing	0	0	155 291 (57.2)	9748 (67.2)
Parental educational level				
Compulsory up to 9 y	77 418 (7.7)	2975 (7.1)	19 833 (7.5)	1119 (7.8)
Secondary	569 588 (56.6)	24 281 (58.0)	137 589 (51.7)	7484 (52.5)
Postsecondary	358 589 (35.7)	14 610 (34.9)	108 595 (40.8)	5658 (39.7)
Missing	36 449 (3.5)	1114 (2.6)	5369 (2.0)	254 (1.7)
Parental occupational classification				
Low	454 686 (46.8)	19 119 (47.0)	101 348 (39.0)	5383 (38.7)
Intermediate	204 987 (21.1)	8862 (21.8)	57 228 (22.0)	3234 (23.2)
High	312 024 (32.1)	12 735 (31.3)	101 156 (38.9)	5296 (38.1)
Missing	70 347 (6.8)	2264 (5.3)	11 654 (4.3)	602 (4.1)
Maternal marital status				
Unmarried	531 164 (51.2)	23 683 (55.3)	69 397 (25.6)	3574 (24.7)
Married	475 964 (45.9)	18 011 (42.1)	188 619 (69.6)	10 244 (70.7)
Divorced or widowed	29 539 (2.8)	1136 (2.7)	12 854 (4.7)	670 (4.6)
Missing	5377 (0.5)	150 (0.3)	516 (0.2)	27 (0.2)
Offspring sex				
Male	534 393 (51.3)	22 519 (52.4)	271 386 (100)	14 515 (100)
Female	507 651 (48.7)	20 461 (47.6)	0	0
Offspring birth weight, kg				
<2.50	28 716 (2.8)	7381 (17.2)	6358 (2.4)	1498 (10.4)
2.50–3.99	813 138 (78.2)	29 635 (69.1)	203 855 (75.5)	10 190 (70.6)
≥4.00	198 274 (19.1)	5846 (13.6)	59 868 (22.2)	2747 (19.0)
Mean (SD)	3549.3 (539.8)	3193.6 (801.8)	3604.2 (534.0)	3397.2 (712.7)
Missing	1916 (0.2)	118 (0.3)	1305 (0.5)	80 (0.6)
Offspring gestational age at birth, wk				
28-31	5201 (0.5)	1496 (3.5)	1063 (0.4)	181 (1.3)
32-36	41 769 (4.0)	6134 (14.3)	11 966 (4.4)	1520 (10.5)
37-41	918 582 (88.3)	33 399 (77.8)	237 002 (87.6)	12 018 (83.0)
≥42	74 764 (7.2)	1878 (4.4)	20 410 (7.5)	757 (5.2)
Mean (SD)	39.4 (1.8)	38.3 (2.7)	39.4 (1.7)	38.8 (2.2)
Missing	1728 (0.2)	73 (0.2)	945 (0.3)	39 (0.3)

Abbreviations: BMI, body mass index (calculated as weight in kilograms divided by height in meters squared); HDP, hypertensive disorders of pregnancy.

^a^ For all variables, No. (%) and mean (SD) values are given only for offspring with no missing values to facilitate comparison by maternal HDP.

### Neurodevelopmental Disorders

**Figure 1.**
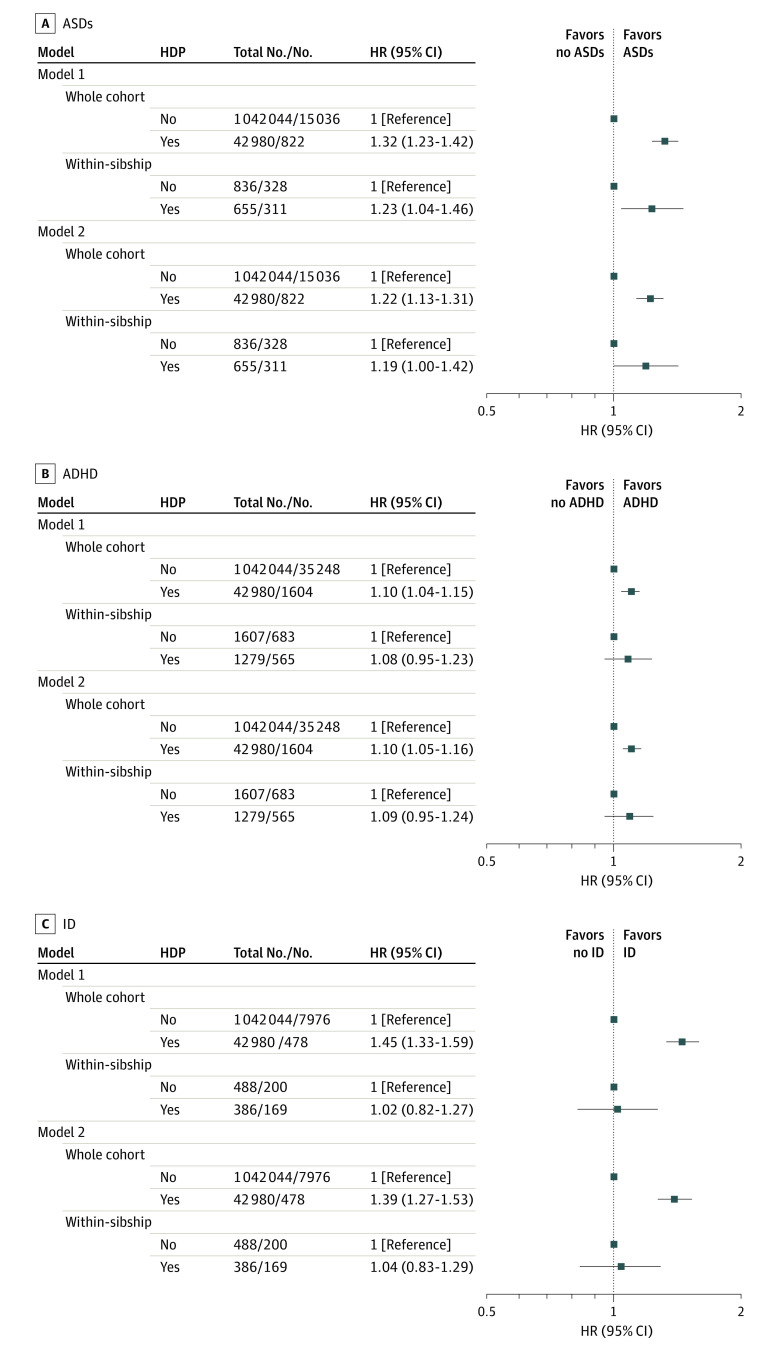
Associations Between Hypertensive Disorders of Pregnancy (HDP) and Risk of Neurodevelopmental Disorders in Offspring Hazard ratios (HRs) for autism spectrum disorders (ASDs), attention-deficit/hyperactivity disorder (ADHD), and intellectual disability (ID) in the 1987-1996 birth cohort comparing offspring born to mothers with HDP with those born to mothers without HDP (reference category). Model 1: adjusted for calendar year of birth only. Model 2: adjusted for calendar year of birth, offspring sex, maternal age, parity, height, body mass index, smoking, pregestational diabetes, parental educational level, occupation, and marital status. The within-sibship models include offspring with at least 1 sibling and compare siblings discordant for maternal HDP, including all covariates listed in whole-cohort analyses except for maternal height and marital status (model 2) because these variables did not vary among siblings within the same family. Number of within-sibship analyses refers to the number of offspring with siblings discordant for maternal HDP and ASDs, ADHD, and ID, respectively.

In the 1987-1996 birth cohort, offspring were followed up until a median age of 22.8 years (interquartile range, 20.3-25.2 years); 15 858 individuals were identified as having ASDs, 36 852 as having ADHD, and 8454 as having ID. In whole-cohort analysis with adjustment for calendar year of birth, HDP were associated with a 1.3-fold increased incidence of ASDs (hazard ratio [HR], 1.32; 95% CI, 1.23-1.42) and a 1.1-fold increased incidence of ADHD (HR, 1.10; 95% CI, 1.04-1.15) (Figure 1). Adjustment for all measured confounders attenuated the estimate for ASDs (HR, 1.22; 95% CI, 1.13-1.31) but not ADHD (HR, 1.10; 95% CI, 1.05-1.16). Analysis using sibling comparisons produced estimates of similar magnitude, but with wider 95% CIs (ASDs: HR, 1.19; 95% CI, 1.00-1.42; ADHD: HR, 1.09; 95% CI, 0.95-1.24). Hypertensive disorders of pregnancy were also associated with an increased incidence of ID among offspring in the whole cohort (HR, 1.39; 95% CI, 1.27-1.53) but not when comparing siblings (HR, 1.04; 95% CI, 0.83-1.29). Analyses using a stricter case definition did not notably change the observed associations with each neurodevelopmental disorder (eTable 2 in the Supplement). Of the 15 858 individuals with diagnoses of ASDs, only 2565 (16.2%) also had ID. In whole-cohort and within-sibship analyses, the association between HDP and ASDs was of higher magnitude for ASDs with comorbid ID compared with ASDs without ID (eTable 3 in the Supplement).

Additional adjustment for gestational age and birth weight for gestational age did not change the HRs for ASDs and ADHD (eTable 4 in the Supplement). The HR for ID was strongly attenuated with additional adjustment for these variables in the whole cohort (HR, 1.15; 95% CI, 1.05-1.27), but a similar null association was found when comparing siblings. Analyses stratified by offspring sex showed associations of similar magnitude with ASDs and ADHD for girls and boys. The association with ID in whole-cohort analysis was of somewhat higher magnitude for girls, but statistical power in this and the corresponding within-sibship analysis was limited (eTable 5 in the Supplement).

### Overall Cognitive Performance

**Figure 2.**
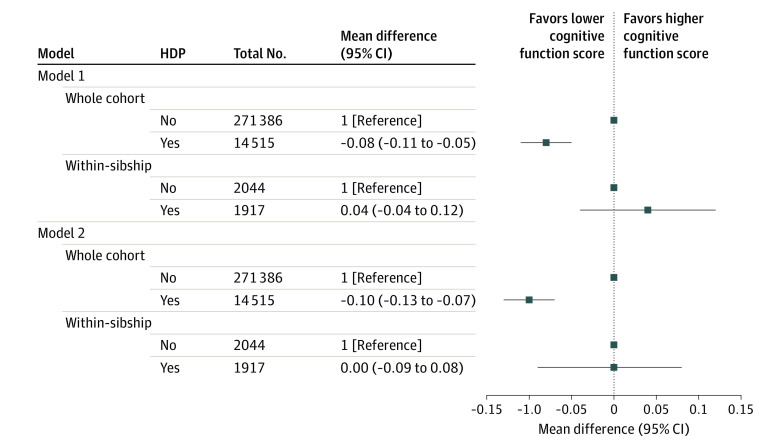
Association Between Hypertensive Disorders of Pregnancy (HDP) and Overall Cognitive Performance in Offspring Mean differences in cognitive function score in the military conscription cohort comparing offspring born to mothers with HDP with those born to mothers without HDP (reference category). Model 1: adjusted for calendar year of birth only. Model 2: adjusted for calendar year of birth, offspring sex, maternal age, parity, height, body mass index, smoking, pregestational diabetes, parental educational level, occupation, and marital status. The within-sibship models include offspring with at least 1 sibling and compare siblings discordant for maternal HDP, including all covariates listed in whole-cohort analyses except for maternal height and marital status (model 2) because these variables did not vary among siblings within the same family. Number of within-sibship analyses refers to the number of offspring with siblings discordant for maternal HDP and cognitive function score.

The mean (SD) cognitive function score in the military conscription cohort was 5.1 (1.9). Offspring exposed to HDP had a slightly lower score compared with those unexposed after adjustment for all measured confounders in whole-cohort analysis (mean difference, −0.10; 95% CI, −0.13 to −0.07). In the within-sibship analysis, the point estimate indicated a null association (mean difference, 0.00; 95% CI, −0.09 to 0.08) (Figure 2). Results for cognitive function assessed as an ordinal outcome demonstrated no evidence of a nonlinear trend in whole-cohort analyses. In equivalent within-sibship analysis, there was some nonlinearity in the association with HDP, although 95% CIs were wide and included the null for lower and higher scores (eTable 6 in the Supplement).

### Sensitivity Analyses

Overall, effect estimates for all neurodevelopmental outcomes were not altered notably by varying conditions in sensitivity analyses (eTables 7-14 in the Supplement), although analyses by calendar period of birth and between-sibling age difference were characterized by limited power. Results were also not materially different in complete case analyses, but the within-sibship association with ADHD was not observed with adjustment for maternal BMI in multivariable-adjusted analyses (eTable 15 in the Supplement).

## Discussion

In conventional analyses adjusted for measured confounding factors, we found that offspring who experienced HDP in utero had a small increased incidence of ASDs, ADHD, and ID and somewhat lower mean cognitive scores at 18 years of age compared with offspring who did not experience HDP in utero. In within-sibship analyses, which account for confounding by unmeasured shared familial characteristics, associations of similar magnitude were observed for ASDs and ADHD, although effect estimates (for ADHD in particular) did not reach conventional *P* value thresholds deemed statistically significant. No clear evidence of an association was found for ID and overall cognitive performance in within-sibship analyses. Together, these results suggest that, while modestly increased risks of ASDs, and possibly ADHD, may be explained by intrauterine effects of HDP, associations with ID and cognitive performance are likely confounded by unmeasured shared familial (environmental or genetic) factors.

### Comparison With Other Studies

The magnitude of the associations observed with ASDs and ADHD is somewhat lower than those reported in a meta-analysis summarizing results of conventional whole-cohort analyses,^4^ showing an approximately 30% increased incidence of ASDs and ADHD associated with HDP. However, studies included in this meta-analysis had limited control for confounding factors, including socioeconomic, lifestyle, and genetic characteristics. More recent studies with better confounder control evaluating associations with preeclampsia only^29,30,31^ have reported effect estimates similar to those found in our study for HDP, including evidence of lower-magnitude associations for ADHD than ASDs. Our within-sibship results are also consistent with findings from a study by Leppert et al^19^ showing that maternal risk alleles for ASDs and ADHD are not associated with HDP. However, in that same study, maternal genetic predisposition to ADHD was associated with a range of other prenatal exposures, including maternal BMI. Because maternal BMI was not routinely measured for all individuals in our study, this might have biased the within-sibship association with ADHD observed in complete case analyses. Adjustment for gestational age and birth weight for gestational age did not materially change the association, indicating that impaired fetal growth is not implicated in the putative associations. Other mechanisms may include reduced fetal oxygenation resulting in structural brain alterations^32^ or inflammatory conditions that have been associated with both HDP^33^ and neurodevelopmental outcomes in offspring.^34^

Previous systematic reviews point to a possible positive association of HDP with ID and an inverse association with overall cognitive performance, but they highlight that this evidence comes mostly from studies involving selected (rather than general) populations, such as those with low birth weight or born preterm, and studies that often fail to adjust for maternal characteristics that are likely to confound the association.^4,15^ A moderately large population-based study of children born in the 1950s in Scotland that adjusted for a wide range of potential confounders (including familial factors) found no association with offspring’s intelligence score measured in childhood.^35^ Between-study heterogeneous results may also be the consequence of the different methods used to assess these outcomes across studies. A cohort study of male conscripts in Finland found a lower cognitive score for those born to mothers with HDP.^11^ However, in our study, the magnitude of association was much smaller in whole-cohort analyses, and we failed to replicate this association in within-sibship analyses.

### Implications of the Research

The consistent finding of an association of maternal HDP with ASDs, and possibly ADHD, in offspring suggests that HDP may represent an early-life risk factor for these outcomes. However, the magnitude of these associations might be too weak (for ADHD in particular) to be considered an important risk factor at the level of the general population, although it is anticipated that an increasing number of women will enter their pregnancy with high blood pressure.^36,37^ Therefore, further research is warranted in this area, ideally with more specific definitions of HDP and ASDs. The lack of within-sibship associations for maternal HDP with offspring ID and overall cognitive performance suggests that the intrauterine milieu of HDP-complicated pregnancies does not play a critical role in the development of these outcomes.

### Strengths and Limitations

Our study has some strengths, including a population-based design with diagnoses obtained using individual record linkage from national registers, minimizing selection and information bias. We further examined associations across a broad spectrum of cognitive performance, including the extreme end of receiving a diagnosis of ID.

Several limitations are also noteworthy. First, we did not have diagnostic data from outpatient specialist records or psychometric measures not covered by the National Patient Register. A high positive predictive value has been reported for ASD diagnoses in this register (94%),^38^ but no external validation data exist for other outcomes. Register-based diagnoses are likely to be more conservative, and differential ascertainment due to socioeconomic factors and family history (including parental mental illness) may exist. However, because these factors tend to cluster within families, within-sibship analysis, at least to some extent, addresses this potential bias. Second, cognitive score data were available for men who were assessed for conscription into military service; most of these men were in good health, so those with an intellectual impairment may not have been included. Hence, results for this outcome may not be generalizable to the total Swedish population. Third, we had no data that allowed us to study associations with specific HDP diagnoses. Currently, there is insufficient evidence as to whether associations with neurodevelopmental outcomes are different for gestational hypertension and preeclampsia,^4,15^ partly because of the variable criteria used by studies to distinguish these diagnoses. The Medical Birth Register also does not hold information on gestational blood pressure and antihypertensive medications. Further studies investigating the influence of blood pressure control and treatment on the observed associations are therefore warranted. Fourth, within-sibship analyses are more susceptible to confounding by individual-level factors (as opposed to family-level confounders).^39^ We aimed to reduce potential bias by adjusting the within-sibship analyses for measured confounders used in the whole cohort that can differ between siblings of the same family.^40,41^ The actual familial discordance in HDP may also be less than modeled, but the impact of this is anticipated to be the same for the different outcomes studied. Fifth, within-sibship analyses are less statistically powered, and we carefully interpreted these results, focusing on the magnitude of associations as compared with those observed in whole-cohort analyses. Also, considering the relatively low incidence of ID and the smaller cohort for cognitive performance analysis, we cannot completely rule out associations for these 2 outcomes, but these would be very small given the effect estimates observed, and the clinical relevance of any such associations is likely to be limited.

## Conclusions

Maternal HDP appear to be associated with small increased risks of ASDs and possibly ADHD in offspring. Associations with ID and overall cognitive performance are likely due to confounding by shared familial factors.

## Notes

Supplement. eMethods. eReferences. eFigure. Flow Charts of the Study Cohorts for Analysis eTable 1. *International Classification of Diseases* Codes for Exposure, Outcomes and Covariates Included in Analyses eTable 2. Associations Between Maternal Hypertensive Disorders of Pregnancy and Risk of Neurodevelopmental Disorders in Offspring – Analyses Including Cases Based on Two Repeat Diagnoses eTable 3. Associations Between Maternal Hypertensive Disorders of Pregnancy and Risk of Autism Spectrum Disorders With and Without Comorbid Intellectual Disability in Offspring eTable 4. Associations Between Maternal Hypertensive Disorders of Pregnancy and Neurodevelopmental Outcomes in Offspring – Analyses With Additional Adjustment for Gestational Age at Birth and Birth Weight for Gestational Age eTable 5. Associations Between Maternal Hypertensive Disorders of Pregnancy and Risk of Neurodevelopmental Disorders in Offspring – Analyses Stratified by Offspring Sex eTable 6. Association Between Maternal Hypertensive Disorders of Pregnancy and Overall Cognitive Performance in Offspring – Results From Multinomial Logistic Regression Analyses eTable 7. Associations Between Maternal Hypertensive Disorders of Pregnancy and Neurodevelopmental Outcomes in Offspring – Whole Cohort Analyses in Offspring With and Without Siblings eTable 8. Associations Between Maternal Hypertensive Disorders of Pregnancy and Neurodevelopmental Outcomes in Offspring – Analyses With Additional Adjustment for Other Conditions Complicating Pregnancy, Birth or the Puerperium eTable 9. Associations Between Maternal Hypertensive Disorders of Pregnancy and Risk of Neurodevelopmental Disorders in Offspring – Analyses Stratified by Calendar Period of Birth eTable 10. Association Between Maternal Hypertensive Disorders of Pregnancy and Overall Cognitive Performance in Offspring– Analyses Stratified by Calendar Period of Birth eTable 11. Associations Between Maternal Hypertensive Disorders of Pregnancy and Neurodevelopmental Outcomes in Offspring – Within-Sibship Analyses Including Full Siblings Only eTable 12. Associations Between Maternal Hypertensive Disorders of Pregnancy and Risk of Neurodevelopmental Disorders in Offspring – Within-Sibship Analyses Stratified by Between-Sibling Age Difference eTable 13. Associations Between Maternal Hypertensive Disorders of Pregnancy and Overall Cognitive Performance in Offspring – Within-Sibship Analyses Stratified by Between-Sibling Age Difference eTable 14. Associations Between Maternal Hypertensive Disorders of Pregnancy and Neurodevelopmental Outcomes in Offspring – Analyses by Birth Order of HDP Occurrence eTable 15. Associations Between Maternal Hypertensive Disorders of Pregnancy and Neurodevelopmental Outcomes in Offspring – Complete Case Analyses
