# Supplementary material for: Association Between Hypertensive Disorders of Pregnancy and Neurodevelopmental Outcomes Among Offspring
Source: JAMA Pediatr. 2021 Mar 22;175(6):1–9. doi: 10.1001/jamapediatrics.2020.6856 (PMC7985818; doi:10.1001/jamapediatrics.2020.6856)
Supplement: Supplement. — eMethods. eReferences. eFigure. Flow Charts of the Study Cohorts for Analysis eTable 1. International Classification of Diseases Codes for Exposure, Outcomes and Covariates Included in Analyses eTable 2. Associations Between Maternal Hypertensive Disorders of Pregnancy and Risk of Neurodevelopmental Disorders in Offspring – Analyses Including Cases Based on Two Repeat Diagnoses eTable 3. Associations Between Maternal Hypertensive Disorders of Pregnancy and Risk of Autism Spectrum Disorders With and Without Comorbid Intellectual Disability in Offspring eTable 4. Associations Between Maternal Hypertensive Disorders of Pregnancy and Neurodevelopmental Outcomes in Offspring – Analyses With Additional Adjustment for Gestational Age at Birth and Birth Weight for Gestational Age eTable 5. Associations Between Maternal Hypertensive Disorders of Pregnancy and Risk of Neurodevelopmental Disorders in Offspring – Analyses Stratified by Offspring Sex eTable 6. Association Between Maternal Hypertensive Disorders of Pregnancy and Overall Cognitive Performance in Offspring – Results From Multinomial Logistic Regression Analyses eTable 7. Associations Between Maternal Hypertensive Disorders of Pregnancy and Neurodevelopmental Outcomes in Offspring – Whole Cohort Analyses in Offspring With and Without Siblings eTable 8. Associations Between Maternal Hypertensive Disorders of Pregnancy and Neurodevelopmental Outcomes in Offspring – Analyses With Additional Adjustment for Other Conditions Complicating Pregnancy, Birth or the Puerperium eTable 9. Associations Between Maternal Hypertensive Disorders of Pregnancy and Risk of Neurodevelopmental Disorders in Offspring – Analyses Stratified by Calendar Period of Birth eTable 10. Association Between Maternal Hypertensive Disorders of Pregnancy and Overall Cognitive Performance in Offspring– Analyses Stratified by Calendar Period of Birth eTable 11. Associations Between Maternal Hypertensive Disorders of Pregnancy and Neurodevelopmental Outcom [file jamapediatr-e206856-s001.pdf]

Supplementary Online Content

Brand JS, Lawlor DA, Larsson H, Montgomery S. Association between hypertensive disorders of pregnancy and neurodevelopmental outcomes among offspring. *JAMA Pediatr.* Published online March 22, 2021. doi:10.1001/jamapediatrics.2020.6856

eMethods.
eReferences.
eFigure. Flow Charts of the Study Cohorts for Analysis
eTable 1. *International Classification of Diseases* Codes for Exposure, Outcomes and Covariates Included in Analyses
eTable 2. Associations Between Maternal Hypertensive Disorders of Pregnancy and Risk of Neurodevelopmental Disorders in Offspring – Analyses Including Cases Based on Two Repeat Diagnoses
eTable 3. Associations Between Maternal Hypertensive Disorders of Pregnancy and Risk of Autism Spectrum Disorders With and Without Comorbid Intellectual Disability in Offspring
eTable 4. Associations Between Maternal Hypertensive Disorders of Pregnancy and Neurodevelopmental Outcomes in Offspring – Analyses With Additional Adjustment for Gestational Age at Birth and Birth Weight for Gestational Age
eTable 5. Associations Between Maternal Hypertensive Disorders of Pregnancy and Risk of Neurodevelopmental Disorders in Offspring – Analyses Stratified by Offspring Sex
eTable 6. Association Between Maternal Hypertensive Disorders of Pregnancy and Overall Cognitive Performance in Offspring – Results From Multinomial Logistic Regression Analyses
eTable 7. Associations Between Maternal Hypertensive Disorders of Pregnancy and Neurodevelopmental Outcomes in Offspring – Whole Cohort Analyses in Offspring With and Without Siblings
eTable 8. Associations Between Maternal Hypertensive Disorders of Pregnancy and Neurodevelopmental Outcomes in Offspring – Analyses With Additional Adjustment for Other Conditions Complicating Pregnancy, Birth or the Puerperium
eTable 9. Associations Between Maternal Hypertensive Disorders of Pregnancy and Risk of Neurodevelopmental Disorders in Offspring – Analyses Stratified by Calendar Period of Birth
eTable 10. Association Between Maternal Hypertensive Disorders of Pregnancy and Overall Cognitive Performance in Offspring– Analyses Stratified by Calendar Period of Birth
eTable 11. Associations Between Maternal Hypertensive Disorders of Pregnancy and Neurodevelopmental Outcomes in Offspring – Within-Sibship Analyses Including Full Siblings Only
eTable 12. Associations Between Maternal Hypertensive Disorders of Pregnancy and Risk of Neurodevelopmental Disorders in Offspring – Within-Sibship Analyses Stratified by Between-Sibling Age Difference
eTable 13. Associations Between Maternal Hypertensive Disorders of Pregnancy and Overall Cognitive Performance in Offspring – Within-Sibship Analyses Stratified by Between-Sibling Age Difference
eTable 14. Associations Between Maternal Hypertensive Disorders of Pregnancy and Neurodevelopmental Outcomes in Offspring – Analyses by Birth Order of HDP Occurrence
eTable 15. Associations Between Maternal Hypertensive Disorders of Pregnancy and Neurodevelopmental Outcomes in Offspring – Complete Case Analyses

© 2021 Brand JS et al. *JAMA Pediatrics*.

This supplementary material has been provided by the authors to give readers additional information about their work.

© 2021 Brand JS et al. *JAMA Pediatrics*.

eMethods.

Description of Swedish registers used in this study

For this study, the data are from national Swedish registers. Each resident in Sweden is assigned a unique personal identification number, which is included in all registers and allows data linkage across the registers. The Medical Birth Register[1] includes maternal, obstetric and neonatal data for > 98% of all births in Sweden since 1973 including maternal diagnoses during pregnancy and child birth coded according to Swedish versions of the International Classification of Diseases (i.e. ICD-8 from 1969 to 1986 and ICD-9 from 1987 to 1996). The Multi-Generation Register[2] includes information on familial relationships for all individuals born since 1932, such that siblings, parents and offspring can be identified. The Total Population Register[3] includes dates of birth, death, and migration since 1915. The Cause of Death Register[4] is a virtually complete register of all deaths in Sweden since 1961. The National Patient Register[5] was established in 1964 and has covered all inpatient hospital admissions since 1987 and outpatient hospital visits to physicians since 2001. Diagnoses in the National Patient Register are coded according to Swedish versions of the International Classification of Diseases (i.e. ICD-9 from 1987 to 1996 and ICD-10 from 1997 onwards), and have been demonstrated to be of high validity with a reported positive predictive value for most diagnoses of 85-95%.[5] Socioeconomic variables are registered in LISA (Longitudinal Integration Database for Health Insurance and Labour Market Studies),[6] which is managed by Statistics Sweden. Using these data sources, we identified 1,085,024 singletons born in Sweden between 1987 and 1996 with data on maternal hypertensive disorders of pregnancy (HDP) and who were followed up until December 31st, 2014. This cohort was used for examining associations of maternal HDP with offspring risk of autism spectrum disorders, attention-deficit/hyperactivity disorder and intellectual disability. For the sibling comparison analysis, we used a subset of the 1987-1996 birth cohort, comprising 716,521 individuals born to 324,713 mothers identified through linkage to the Multi-Generation Register.

The association of maternal HDP with overall cognitive performance in offspring was explored in a cohort including 295,448 male singletons born in Sweden between 1982 and 1992, with available data on cognitive function scores at the first military assessment, as identified through linkage to the Swedish Military Conscription Register. In Sweden, military conscription was mandatory for men at the age of 18 years, but from 2002/2003 the number of conscripts declined substantially due to reduced military needs, and conscription was officially suspended in 2010. Among this military conscription cohort, we identified 70,642 siblings born to 34,157 mothers based on linkage to the Multi-Generation Register. During the study period, different cognitive function tests were used as described in detail elsewhere.[7] Briefly, between 1982 and 1994 a written test was used, and after 1994 a computer-based test. In both tests a global cognitive function score was obtained based on subtasks covering logical, spatial, verbal and technical abilities, and this score was standardised against the entire tested population to follow a Gaussian distribution with values between 1 and 9.

Handling of missing covariate data

In total, 507,479 offspring (46.8%) in the 1987-1996 birth cohort and 131,861 offspring (46.1%) in the military conscription cohort had missing data on one or more covariates, which was mainly due to missing information on early pregnancy maternal BMI and height. This is because maternal weight and height were not routinely recorded in women who were pregnant before 1992. Missing covariate data were imputed under the 'missing at random assumption' using multivariate multiple imputation with chained equations,[8] and 5 imputed datasets were generated. The imputation model included the exposure (HDP), the outcome (for autism spectrum disorders, attention-deficit/hyperactivity disorder and intellectual disability the Nelson-Aalen estimator of the baseline cumulative hazard and event indicator for these outcomes), all covariates included in the multivariable adjusted model (i.e. birth year, offspring sex, maternal age, parity, height, body mass index, smoking, pregestational diabetes, parental education, occupation, and marital status). Main analyses were based on imputed data, but we also conducted sensitivity analyses with complete case data only.

eReferences

1. National Board of Health and Welfare. The Swedish Medical Birth Register - A summary of Content and Quality. Stockholm: Centre for Epidemiology, 2003. https://www.socialstyrelsen.se/globalassets/sharepoint-dokument/artikelkatalog/ovrigt/2003-112-3_20031123.pdf Accessed May 2020.
2. Ekbom A. The Swedish Multi-generation Registe. In: *Methods Mol Biol*.; 2011:215-220. doi:10.1007/978-1-59745-423-0_10
3. Ludvigsson JF, Almqvist C, Bonamy AKE, et al. Registers of the Swedish total population and their use in medical research. *Eur J Epidemiol*. 2016;31(2):125-136. doi:10.1007/s10654-016-0117-y
4. Brooke HL, Talbäck M, Hörnblad J, et al. The Swedish cause of death register. *Eur J Epidemiol*. 2017;32(9):765-773. doi:10.1007/s10654-017-0316-1
5. Ludvigsson JF, Ekbom A, Kim J-L, et al. External review and validation of the Swedish national

© 2021 Brand JS et al. *JAMA Pediatrics*.

inpatient register. *BMC Public Health*. 2011;11(1):450. doi:10.1186/1471-2458-11-450
6. Ludvigsson JF, Svedberg P, Olén O, Bruze G, Neovius M. The longitudinal integrated database for health insurance and labour market studies (LISA) and its use in medical research. *Eur J Epidemiol*. 2019;34(4):423-437. doi:10.1007/s10654-019-00511-8
7. Wennerstad KM, Silventoinen K, Tynelius P, Bergman L, Kaprio J, Rasmussen F. Associations between IQ and cigarette smoking among Swedish male twins. *Soc Sci Med*. 2010;70(4):575-581. doi:10.1016/j.socscimed.2009.10.050
8. White IR, Royston P. Imputing missing covariate values for the Cox model. *Stat Med* 2009;28:1982-98 doi:10.1002/sim.3618.

© 2021 Brand JS et al. *JAMA Pediatrics*.

eFigure. Flow Charts of the Study Cohorts for Analysis

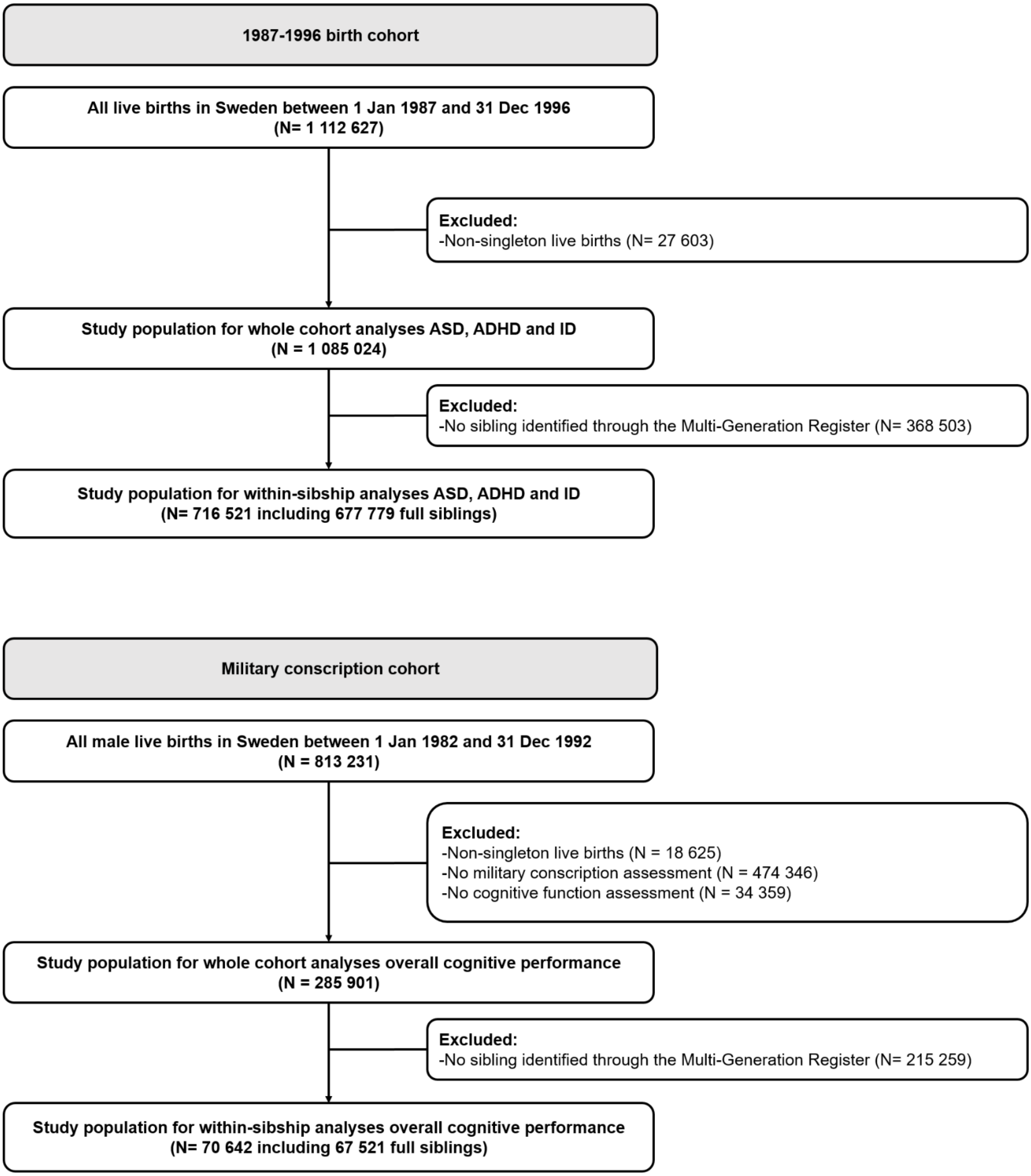

Associations of HDP with autism spectrum disorders, attention-deficit/hyperactivity disorder and intellectual disability were studied in the 1987-1996 birth cohort. Associations of HDP with cognitive function in the normal range was studies in the military conscription cohort.
Abbreviations: ASD = autism spectrum disorders; ADHD = attention-deficit/hyperactivity disorder; ID = intellectual disability.

© 2021 Brand JS et al. *JAMA Pediatrics*.

eTable 1. *International Classification of Diseases* Codes for Exposure, Outcomes and Covariates Included in Analyses

Variable	Source	Definition	ICD code
Exposure	Medical Birth Register	Hypertension disorders of pregnancy	ICD-9 (642), ICD-8 (401, 637)
Outcome	National Patient Register	Autism spectrum disorders	ICD-10 (F84), ICD-9 (299)
Outcome	National Patient Register	Attention-deficit hyperactivity disorder	ICD-10 (F90), ICD-9 (314)
Outcome	National Patient Register	Intellectual disability	ICD-10 (F70-79), ICD-9 (317-319)
Covariate	Medical Birth Register	Maternal pregestational diabetes	ICD-9 (250), ICD-8 (250)
Covariate	Medical Birth Register	Other conditions complicating pregnancy, childbirth or the puerperium (gestational diabetes, thyroid dysfunction, anaemia, drug dependency, mental disorders, congenital cardiovascular diseases, other cardiovascular diseases, bone and joint disorders of back pelvis and lower limbs)	ICD-9 (648) [a]

[a] Information on other conditions complicating pregnancy, childbirth or the puerperium was only available for offspring with ICD-9 diagnostic code data, as equivalent codes do not exist in the ICD-8 chapter.
Abbreviations: ICD = international classification of diseases.

© 2021 Brand JS et al. *JAMA Pediatrics*.

eTable 2. Associations Between Maternal Hypertensive Disorders of Pregnancy and Risk of Neurodevelopmental Disorders in Offspring – Analyses Including Cases Based on Two Repeat Diagnoses

	**Autism spectrum disorders**		**Attention-deficit hyperactivity disorder**		**Intellectual disability**	
	N total / N ASD	HR (95% CI)	N total / N ADHD	HR (95% CI)	N total / N ID	HR (95% CI)
**MODEL 1**						
**Whole cohort**						
No HDP	1042044 / 8481	REF	1042044 / 21857	REF	1042044 / 4302	REF
HDP	42980 / 493	1.41 (1.28; 1.54)	42980 / 1013	1.12 (1.05; 1.19)	42980 / 258	1.45 (1.28; 1.65)
**Within-sibship**						
No HDP	422 / 157	REF	1033 / 450	REF	251 / 112	REF
HDP	345 / 165	1.30 (1.04; 1.61)	828 / 357	1.10 (0.94; 1.28)	201 / 83	0.96 (0.71; 1.28)
**MODEL 2**						
**Whole cohort**						
No HDP	1042044 / 8481	REF	1042044 / 21857	REF	1042044 / 4302	REF
HDP	42980 / 493	1.28 (1.17; 1.41)	42980 / 1013	1.12 (1.05; 1.20)	42980 / 258	1.39 (1.23; 1.58)
**Within-sibship**						
No HDP	473 / 179	REF	1033 / 450	REF	251 / 112	REF
HDP	382 / 186	1.26 (1.01; 1.58)	828 / 357	1.10 (0.94; 1.28)	201 / 83	0.95 (0.70; 1.29)

Hazard ratios for autism spectrum disorders, attention-deficit hyperactivity disorder and intellectual disability in the 1987-1996 birth cohort comparing offspring born to mothers with HDP with those born to mothers without HDP (= reference category) – analysis including cases based on two repeat diagnoses.
Model 1: adjusted for calendar year of birth only.
Model 2: adjusted for calendar year of birth, offspring sex, maternal age, parity, height, body mass index, smoking, pregestational diabetes, parental education, occupation and marital status.
The within-sibship models include offspring with at least 1 sibling and compare siblings discordant for maternal HDP, including all covariates listed in whole cohort analyses except for maternal height and marital status (model 2), as these variables were among siblings within the same family.
N of within-sibship analyses refers to the number of offspring with siblings discordant on maternal HDP and each neurodevelopmental outcome.
Abbreviations: ASD = autism spectrum disorders; ADHD = attention-deficit hyperactivity disorder**;** ID = intellectual disability; HDP = hypertensive disorders of pregnancy; HR = hazard ratio; CI = confidence interval.

© 2021 Brand JS et al. *JAMA Pediatrics*.

eTable 3. Associations Between Maternal Hypertensive Disorders of Pregnancy and Risk of Autism Spectrum Disorders With and Without Comorbid Intellectual Disability in Offspring

	**Autism spectrum disorders with intellectual disability**		**Autism spectrum disorders without intellectual disability**	
	N total / N ASD with ID	HR (95% CI)	N total / N ASD with ID	HR (95% CI)
**MODEL 1**				
**Whole cohort**				
No HDP	1042044 / 2406	REF	1042044 / 12630	REF
HDP	42980 / 159	1.60 (1.36; 1.88)	42980 / 663	1.27 (1.17; 1.37)
**Within-sibship**				
No HDP	129 / 47	REF	728 / 284	REF
HDP	109 / 55	1.55 (1.01; 2.36)	564 / 260	1.17 (0.97; 1.41)
**MODEL 2**				
**Whole cohort**				
No HDP	1042044 / 2406	REF	1042044 / 12630	REF
HDP	42980 / 159	1.52 (1.29; 1.79)	42980 / 663	1.16 (1.07; 1.26)
**Within-sibship**				
No HDP	129 / 47	REF	728 / 284	REF
HDP	109 / 55	1.70 (1.10; 2.63)	564 / 260	1.10 (0.90; 1.33)

Hazard ratios for autism spectrum disorders with and without intellectual disability in the 1987-1996 birth cohort comparing offspring born to mothers with HDP with those born to mothers without (= reference category).
Model 1: adjusted for calendar year of birth only.
Model 2: adjusted for calendar year of birth, offspring sex, maternal age, parity, height, body mass index, smoking, pregestational diabetes, parental education, occupation and marital status.
The within-sibship models include offspring with at least 1 sibling and compare siblings discordant for maternal HDP, including all covariates listed in whole cohort analyses except for maternal height and marital status (model 2), as these variables did not vary among siblings within the same family.
N of within-sibship analyses refers to the number of offspring with siblings discordant on maternal HDP and each neurodevelopmental outcome.
Abbreviations: ASD = autism spectrum disorders; ID = intellectual disability; HDP = hypertensive disorders of pregnancy; HR = hazard ratio; CI = confidence interval.

© 2021 Brand JS et al. *JAMA Pediatrics*.

eTable 4. Associations Between Maternal Hypertensive Disorders of Pregnancy and Neurodevelopmental Outcomes in Offspring – Analyses With Additional Adjustment for Gestational Age at Birth and Birth Weight for Gestational Age

	**Autism spectrum disorders**		**Attention-deficit hyperactivity disorder**		**Intellectual disability**		**Cognitive function score**	
	N total/N ASD	HR (95% CI)	N total/N ADHD	HR (95% CI)	N total/N ID	HR (95% CI)	N total	Mean difference (95% CI)
**MODEL 3**								
**Whole cohort**								
No HDP	1038487/14974	REF	1038487/35095	REF	1038487/7917	REF	269192	REF
HDP	42792/817	1.15 (1.07; 1.24)	42792/1594	1.05 (1.00; 1.11)	42792/471	1.15 (1.05; 1.27)	14400	-0.08 (-0.11;-0.05)
**Within-sibship**								
No HDP	834/328	REF	1595/677	REF	482/199	REF	2022	REF
HDP	653/309	1.15 (0.96; 1.37)	1267/562	1.07 (0.93; 1.22)	382/166	0.93 (0.74; 1.16)	1896	0.00 (-0.08; 0.09)

Hazard ratios for autism spectrum disorders, attention-deficit hyperactivity disorder and intellectual disability in the 1987-1996 birth cohort and mean difference in cognitive function score in the military conscription cohort comparing offspring born to mothers with HDP with those born to mothers without (= reference category).
Model 3: adjusted for calendar year of birth, offspring sex, maternal age, parity, height, body mass index, smoking, pregestational diabetes, parental education, occupation and marital status, gestational age at birth and birth weight for gestational age.
The within-sibship models include offspring with at least 1 sibling and compare siblings discordant for maternal HDP, including all covariates listed in whole cohort analyses except for maternal height and marital status, as these variables did not vary among siblings within the same family.
N of within-sibship analyses refer to the number of offspring with siblings discordant on maternal HDP and each neurodevelopmental outcome.
Abbreviations: ASD = autism spectrum disorders; ADHD = attention-deficit hyperactivity disorder; ID = intellectual disability; HDP = hypertensive disorders of pregnancy; HR = hazard ratio; CI = confidence interval.

© 2021 Brand JS et al. *JAMA Pediatrics*.

eTable 5. Associations Between Maternal Hypertensive Disorders of Pregnancy and Risk of Neurodevelopmental Disorders in Offspring – Analyses Stratified by Offspring Sex

	**Autism spectrum disorders**			**Attention-deficit hyperactivity disorder**			**Intellectual disability**		
	HR (95% CI)		*P* value	HR (95% CI)		*P* value	HR (95% CI)		*P* value
	Males	Females		Males	Females		Males	Females	
**MODEL 1**									
**Whole cohort**									
No HDP	REF	REF	0.56	REF	REF	0.27	REF	REF	0.04
HDP	1.29 (1.18; 1.41)	1.35 (1.20; 1.52)		1.07 (1.00; 1.14)	1.13 (1.04; 1.22)		1.33 (1.17; 1.51)	1.62 (1.41; 1.86)	
**Within-sibship**									
No HDP	REF	REF	0.95	REF	REF	0.24	REF	REF	0.61
HDP	1.32 (1.00; 1.74)	1.30 (0.88; 1.93)		1.07 (0.92; 1.26)	1.16 (0.94; 1.44)		0.88 (0.59; 1.31)	1.41 (0.91; 2.18)	
**MODEL 2**									
**Whole cohort**									
No HDP	REF	REF	0.56	REF	REF	0.18	REF	REF	0.03
HDP	1.18 (1.08; 1.29)	1.29 (1.14; 1.45)		1.07 (1.00; 1.14)	1.16 (1.07; 1.25)		1.27 (1.12; 1.44)	1.58 (1.38; 1.82)	
**Within-sibship**									
No HDP	REF	REF	0.99	REF	REF	0.25	REF	REF	0.68
HDP	1.29 (0.97; 1.70)	1.28 (0.86; 1.92)		1.08 (0.92; 1.26)	1.15 (0.93; 1.43)		0.87 (0.57; 1.30)	1.48 (0.94; 2.32)	

Hazard ratios for autism spectrum disorders, attention-deficit hyperactivity disorder and intellectual disability in the 1987-1996 birth cohort comparing offspring born to mothers with HDP with those born to mothers without (= reference category), stratified by offspring sex.
Model 1: adjusted for calendar year of birth only.
Model 2: adjusted for calendar year of birth, offspring sex, maternal age, parity, height, body mass index, smoking, pregestational diabetes, parental education, occupation and marital status.
The within-sibship models include offspring with at least 1 sibling and compare siblings discordant for maternal HDP, including all covariates listed in whole cohort analyses except for maternal height and marital status (model 2), as these variables did not vary among siblings within the same family.
P values refer to p values from interaction tests.
Abbreviations: HDP = hypertensive disorders of pregnancy; HR = hazard ratio; CI = confidence interval.

© 2021 Brand JS et al. *JAMA Pediatrics*.

eTable 6. Association Between Maternal Hypertensive Disorders of Pregnancy and Overall Cognitive Performance in Offspring – Results From Multinomial Logistic Regression Analyses

	**Cognitive function score**	
	Low (1-3)	High (7-9)
	RRR (95% CI)	RRR (95% CI)
**MODEL 1**		
**Whole cohort**		
No HDP	REF	REF
HDP	1.06 (1.02; 1.11)	0.93 (0.89; 0.97)
**Within-sibship**		
No HDP	REF	REF
HDP	1.10 (0.93; 1.31)	1.18 (1.00; 1.39)
**MODEL 2**		
**Whole cohort**		
No HDP	REF	REF
HDP	1.09 (1.04; 1.14)	0.91 (0.87; 0.95)
**Within-sibship**		
No HDP	REF	REF
HDP	1.16 (0.98; 1.37)	1.13 (0.96; 1.33)

Relative risk ratios for low and high cognitive function scores in the military conscription cohort comparing offspring born to mothers with HDP with those born to mothers without. All relative risk ratios are from multinomial logistic regression models with scores 4-6 (i.e. medium cognitive function scores) as the reference category.
Model 1: adjusted for calendar year of birth only.
Model 2: adjusted for calendar year of birth, offspring sex, maternal age, parity, height, body mass index, smoking, pregestational diabetes, parental education, occupation and marital status.
The within-sibship models include offspring with at least 1 sibling and compare siblings discordant for maternal HDP, including all covariates listed in whole cohort analyses except for maternal height and marital status (model 2), as these variables did not vary among siblings within the same family.
Abbreviations: RRR = relative risk ratio; CI = confidence interval; HDP = hypertensive disorder of pregnancy.

© 2021 Brand JS et al. *JAMA Pediatrics*.

eTable 7. Associations Between Maternal Hypertensive Disorders of Pregnancy and Neurodevelopmental Outcomes in Offspring – Whole Cohort Analyses in Offspring With and Without Siblings

	**Autism spectrum disorders**		**Attention-deficit hyperactivity disorder**		**Intellectual disability**		**Cognitive function score**	
	N total / N ASD	HR (95% CI)	N total / N ADHD	HR (95% CI)	N total / N ID	HR (95% CI)	N total	Mean difference (95% CI)
**MODEL 1**								
**Offspring without siblings**								
No HDP	350555 / 5405	REF	350555 / 12557	REF	350555 / 2818	REF	203852	REF
HDP	17948 / 381	1.31 (1.18; 1.46)	17948 / 741	1.09 (1.01; 1.18)	17948 / 227	1.56 (1.36; 1.78)	11407	-0.08 (-0.11;-0.04)
**Offspring with siblings [a]**								
No HDP	691489 / 9631	REF	691489 / 22691	REF	691489 / 5158	REF	67534	REF
HDP	25032 / 441	1.29 (1.17; 1.42)	25032 / 863	1.08 (1.01; 1.15)	25032 / 251	1.37 (1.20; 1.55)	3108	-0.04 (-0.11; 0.03)
**MODEL 2**								
**Offspring without siblings**								
No HDP	691489 / 9631	REF	350555 / 12557	REF	350555 / 2818	REF	203852	REF
HDP	25032 / 441	1.21 (1.08; 1.34)	17948 / 741	1.08 (1.01; 1.17)	17948 / 227	1.42 (1.24; 1.63)	11407	-0.09 (-0.13;-0.06)
**Offspring with siblings [a]**								
No HDP	350555 / 5405	REF	691489 / 22691	REF	691489 / 5158	REF	67534	REF
HDP	17948 / 381	1.22 (1.11; 1.35)	25032 / 863	1.11 (1.04; 1.19)	25032 / 251	1.36 (1.20; 1.55)	3108	-0.11 (-0.18;-0.04)

Hazard ratios for autism spectrum disorders, attention-deficit hyperactivity disorder and intellectual disability in the 1987-1996 birth cohort and mean difference in cognitive function score in the military conscription cohort comparing offspring born to mothers with HDP with those born to mothers without (= reference category) – whole cohort analyses in offspring with and without siblings.
Model 1: adjusted for calendar year of birth only.
Model 2: adjusted for calendar year of birth, offspring sex, maternal age, parity, height, body mass index, smoking, pregestational diabetes, parental education, occupation and marital status.
[a] Sandwich estimator corrected standard errors were used to account for familial clustering in the analysis including offspring with siblings.
Abbreviations: ASD = autism spectrum disorders; ADHD = attention-deficit hyperactivity disorder; ID = intellectual disability; HDP = hypertensive disorders of pregnancy; HR = hazard ratio; CI = confidence interval.

© 2021 Brand JS et al. *JAMA Pediatrics*.

eTable 8. Associations Between Maternal Hypertensive Disorders of Pregnancy and Neurodevelopmental Outcomes in Offspring – Analyses With Additional Adjustment for Other Conditions Complicating Pregnancy, Birth or the Puerperium

	**Autism spectrum disorders**		**Attention-deficit hyperactivity disorder**		**Intellectual disability**		**Cognitive function score**	
	N total / N ASD	HR (95% CI)	N total / N ADHD	HR (95% CI)	N total / N ID	HR (95% CI)	N total	Mean difference (95% CI)
**MODEL 4**								
**Whole cohort**								
No HDP	1042044 / 15036	REF	1042044 / 35248	REF	1042044 / 7976	REF	271386	REF
HDP	42980 / 822	1.21 (1.13; 1.30)	42980 / 1604	1.10 (1.04; 1.15)	42980 / 478	1.38 (1.26; 1.52)	14515	-0.10 (-0.13;-0.07)
**Within-sibship**								
No HDP	836 / 328	REF	1607 / 683	REF	488 / 200	REF	2044	REF
HDP	655 / 311	1.19 (1.00; 1.42)	1279 / 565	1.09 (0.95; 1.24)	386 / 169	1.04 (0.83; 1.29)	1917	0.00 (-0.09; 0.08)

Hazard ratios for autism spectrum disorders, attention-deficit hyperactivity disorder and intellectual disability in the 1987-1996 birth cohort and mean difference in cognitive function score in the military conscription cohort of offspring born between 1987 and 1996 comparing offspring born to mothers with HDP with those born to mothers without (= reference category).
Model 4: adjusted for calendar year of birth, offspring sex, maternal age, parity, height, body mass index, smoking, pregestational diabetes, parental education, occupation and marital status, other conditions complicating pregnancy, birth or the puerperium.
The within-sibship models include offspring with at least 1 sibling and compare siblings discordant for maternal HDP, including all covariates listed in whole cohort analyses except for maternal height and marital status, as these variables did not vary among siblings within the same family.
N of within-sibship analyses refer to the number of offspring with siblings discordant on maternal HDP and each neurodevelopmental outcome.
Abbreviations: ASD = autism spectrum disorders; ADHD = attention-deficit hyperactivity disorder; ID = intellectual disability; HDP = hypertensive disorders of pregnancy; HR = hazard ratio; CI = confidence interval.

© 2021 Brand JS et al. *JAMA Pediatrics*.

eTable 9. Associations Between Maternal Hypertensive Disorders of Pregnancy and Risk of Neurodevelopmental Disorders in Offspring – Analyses Stratified by Calendar Period of Birth

	**Autism spectrum disorders**			**Attention-deficit hyperactivity disorder**			**Intellectual disability**		
	HR (95% CI)		*P* value	HR (95% CI)		*P* value	HR (95% CI)		*P* value
	1987-1991	1992-1996		1987-1991	1992-1996		1987-1991	1992-1996	
**MODEL 1**									
**Whole cohort**									
No HDP	REF	REF	0.16	REF	REF	0.14	REF	REF	0.01
HDP	1.24 (1.11; 1.39)	1.38 (1.26; 1.51)		1.15 (1.06; 1.24)	1.06 (1.00; 1.13)		1.27 (1.10; 1.47)	1.61 (1.43; 1.81)	
**Within-sibship**									
No HDP	REF	REF	0.05	REF	REF	0.22	REF	REF	0.17
HDP	1.06 (0.73; 1.53)	1.34 (1.00; 1.78)		1.35 (1.04; 1.74)	1.00 (0.80; 1.25)		0.96 (0.62; 1.49)	1.08 (0.73; 1.59)	
**MODEL 2**									
**Whole cohort**									
No HDP	REF	REF	0.38	REF	REF	0.02	REF	REF	0.05
HDP	1.17 (1.05; 1.31)	1.25 (1.14; 1.37)		1.18 (1.09; 1.28)	1.05 (0.99; 1.13)		1.23 (1.07; 1.43)	1.53 (1.35; 1.73)	
**Within-sibship**									
No HDP	REF	REF	0.09	REF	REF	0.28	REF	REF	0.21
HDP	1.03 (0.70; 1.51)	1.30 (0.96; 1.75)		1.33 (1.02; 1.73)	1.02 (0.81; 1.28)		0.89 (0.57; 1.41)	1.10 (0.74; 1.63)	

Hazard ratios for autism spectrum disorders, attention-deficit hyperactivity disorder and intellectual disability in the 1987-1996 birth cohort comparing offspring born to mothers with HDP with those born to mothers without (= reference category) – sensitivity analysis stratified by calendar period of birth.
Model 1: adjusted for calendar year of birth only.
Model 2: adjusted for calendar year of birth, offspring sex, maternal age, parity, height, body mass index, smoking, pregestational diabetes, parental education, occupation and marital status.
The within-sibship models include offspring with at least 1 sibling and compare siblings discordant for maternal HDP, including all covariates listed in whole cohort analyses except for maternal height and marital status (model 2), as these variables did not vary among siblings within the same family.
P values refer to p values from interaction tests.
Abbreviations: HDP = hypertensive disorders of pregnancy; HR = hazard ratio; CI = confidence interval.

© 2021 Brand JS et al. *JAMA Pediatrics*.

eTable 10. Association Between Maternal Hypertensive Disorders of Pregnancy and Overall Cognitive Performance in Offspring–Analyses Stratified by Calendar Period of Birth

	**Cognitive function score**				
	1982-1986		1987-1992		*P* value
	N total	Mean difference (95% CI)	N total	Mean difference (95% CI)	
**MODEL 1**					
**Whole cohort**					
No HDP	155291	REF	116095	REF	0.72
HDP	9748	-0.07 (-0.11;-0.03)	4767	-0.08 (-0.14;-0.03)	
**Within-sibship**					
No HDP	725	REF	297	REF	0.15
HDP	716	0.11 (-0.03; 0.24)	291	-0.21 (-0.41;-0.01)	
**MODEL 2**					
**Whole cohort**					
No HDP	155291	REF	116095	REF	0.33
HDP	9748	-0.09 (-0.13;-0.05)	4767	-0.12 (-0.17;-0.07)	
**Within-sibship**					
No HDP	725	REF	297	REF	0.14
HDP	716	0.08 (-0.05; 0.22)	291	-0.22 (-0.42;-0.02)	

Mean differences in cognitive function score in the military conscription cohort comparing offspring born to mothers with HDP with those born to mothers without (= reference category) - analyses stratified by calendar period of birth.
Model 1: adjusted for calendar year of birth only.
Model 2: adjusted for calendar year of birth, offspring sex, maternal age, parity, height, body mass index, smoking, pregestational diabetes, parental education, occupation and marital status.
The within-sibship models include offspring with at least 1 sibling and compare siblings discordant for maternal HDP, including all covariates listed in whole cohort analyses except for maternal height and marital status (model 2), as these variables did not vary among siblings within the same family.
N of within-sibship analyses refers to the number of offspring with siblings discordant on maternal HDP and cognitive function score.
P values refer to p values from interaction tests.
Abbreviations: HDP = hypertensive disorders of pregnancy; CI = confidence interval.

© 2021 Brand JS et al. *JAMA Pediatrics*.

eTable 11. Associations Between Maternal Hypertensive Disorders of Pregnancy and Neurodevelopmental Outcomes in Offspring – Within-Sibship Analyses Including Full Siblings Only

	**Autism spectrum disorders**		**Attention-deficit hyperactivity disorder**		**Intellectual disability**		**Cognitive function score**	
	N total / N ASD	HR (95% CI)	N total / N ADHD	HR (95% CI)	N total / N ID	HR (95% CI)	N total	Mean difference (95% CI)
**MODEL 1**								
**Within-sibship**								
No HDP	726 / 287	REF	1322 / 563	REF	432 / 180	REF	1949	REF
HDP	582 / 281	1.19 (1.00; 1.42)	1076 / 487	1.13 (0.98; 1.29)	349 / 153	1.02 (0.82; 1.28)	1826	0.05 (-0.03; 0.14)
**MODEL 2**								
**Within-sibship**								
No HDP	726 / 287	REF	1322 / 563	REF	432 / 180	REF	1949	REF
HDP	582 / 281	1.16 (0.97; 1.40)	1076 / 487	1.11 (0.97; 1.28)	349 / 153	1.04 (0.83; 1.31)	1826	0.02 (-0.07; 0.10)

Hazard ratios for autism spectrum disorders, attention-deficit hyperactivity disorder and intellectual disability in the 1987-1996 birth cohort and mean difference in cognitive function score in the military conscription cohort comparing offspring born to mothers with HDP with those born to mothers without (= reference category) – within-sibship analyses including full siblings only.
Model 1: adjusted for calendar year of birth only.
Model 2: adjusted for calendar year of birth, offspring sex, maternal age, parity, body mass index, smoking and pregestational diabetes. Maternal height, marital status and parental education and occupation in model 2 as these variables did not vary among full siblings within the same family.
N of within-sibship analyses refers to the number of offspring with full siblings discordant on maternal HDP and each neurodevelopmental outcome
Abbreviations: ASD = autism spectrum disorders; ADHD = attention-deficit hyperactivity disorder; ID = intellectual disability; HDP = hypertensive disorders of pregnancy; HR = hazard ratio; CI = confidence interval.

© 2021 Brand JS et al. *JAMA Pediatrics*.

eTable 12. Associations Between Maternal Hypertensive Disorders of Pregnancy and Risk of Neurodevelopmental Disorders in Offspring – Within-Sibship Analyses Stratified by Between-Sibling Age Difference

	**Autism spectrum disorders**			**Attention-deficit hyperactivity disorder**			**Intellectual disability**		
	HR (95% CI)		*P* value	HR (95% CI)		*P* value	HR (95% CI)		*P* value
	Δ age ≤ 3 years	Δ age > 3 years		Δ age ≤ 3 years	Δ age > 3 years		Δ age ≤ 3 years	Δ age > 3 years	
**MODEL 1**									
**Within-sibship**									
No HDP	REF	REF	0.23	REF	REF	0.24	REF	REF	0.96
HDP	1.11 (0.90; 1.37)	1.37 (0.98; 1.90)		1.12 (0.96; 1.30)	0.86 (0.66; 1.12)		1.08 (0.83; 1.41)	0.86 (0.56; 1.32)	
**MODEL 2**									
**Within-sibship**									
No HDP	REF	REF	0.27	REF	REF	0.25	REF	REF	0.94
HDP	1.12 (0.90; 1.39)	1.28 (0.91; 1.79)		1.11 (0.95; 1.30)	0.85 (0.64; 1.11)		1.08 (0.82; 1.41)	0.90 (0.58; 1.40)	

Hazard ratios for autism spectrum disorders, attention-deficit hyperactivity disorder and intellectual disability in the 1987-1996 birth cohort comparing offspring born to mothers with HDP with those born to mothers without (= reference category) – sensitivity analysis stratified by within-sibling age difference.
Model 1: adjusted for calendar year of birth only.
Model 2: adjusted for calendar year of birth, offspring sex, maternal age, parity, body mass index, smoking, pregestational diabetes and parental education.
P values refer to p values from interaction tests.
Abbreviations: HR = hazard ratio; CI = confidence interval.

© 2021 Brand JS et al. *JAMA Pediatrics*.

eTable 13. Associations Between Maternal Hypertensive Disorders of Pregnancy and Overall Cognitive Performance in Offspring – Within-Sibship Analyses Stratified by Between-Sibling Age Difference

	**Cognitive function score**				
	Δ age ≤ 3 years		Δ age > 3 years		*P* value
	N total	Mean difference (95% CI)	N total	Mean difference (95% CI)	
**MODEL 1**					
**Within-sibship**					
No HDP	1169	REF	740	REF	0.19
HDP	1122	0.02 (-0.09; 0.13)	732	0.00 (-0.13; 0.14)	
**MODEL 2**					
**Within-sibship**					
No HDP	1169	REF	740	REF	0.47
HDP	1122	0.00 (-0.11; 0.11)	732	-0.03 (-0.16; 0.10)	

Mean differences in cognitive function score in the military conscription cohort comparing offspring born to mothers with HDP with those born to mothers without (= reference category) – analysis stratified by between-sibling age difference.
Model 1: adjusted for calendar year of birth only.
Model 2: adjusted for calendar year of birth, offspring sex, maternal age, parity, height, body mass index, smoking, pregestational diabetes, parental education, occupation and marital status.
The within-sibship models include offspring with at least 1 sibling and compare siblings discordant for maternal HDP, including all covariates listed in whole cohort analyses except for maternal height and marital status (model 2), as these variables did not vary among siblings within the same family.
N of within-sibship analyses refers to the number of offspring with siblings discordant on maternal HDP and cognitive function score.
P values refer to p values from interaction tests.
Abbreviations: HDP = hypertensive disorders of pregnancy; CI = confidence interval.

© 2021 Brand JS et al. *JAMA Pediatrics*.

eTable 14. Associations Between Maternal Hypertensive Disorders of Pregnancy and Neurodevelopmental Outcomes in Offspring – Analyses by Birth Order of HDP Occurrence

	**Autism spectrum disorders**	**Attention-deficit hyperactivity disorder**	**Intellectual disability**	**Cognitive function score**
	HR (95% CI)	HR (95% CI)	HR (95% CI)	Mean difference (95% CI)
**MODEL 1**				
**Within-sibship**				
No HDP	REF	REF	REF	REF
HDP in nulliparous women	1.23 (0.98; 1.54)	1.10 (0.94; 1.30)	0.88 (0.64; 1.20)	0.09 (-0.01; 0.20)
HDP in parous women	1.24 (0.96; 1.60)	1.07 (0.87; 1.32)	1.18 (0.87; 1.61)	-0.06 (-0.18; 0.07)
**MODEL 2**				
**Within-sibship**				
No HDP	REF	REF	REF	REF
HDP in nulliparous women	1.18 (0.93; 1.50)	1.10 (0.93; 1.30)	0.91 (0.66; 1.25)	0.01 (-0.10; 0.11)
HDP in parous women	1.20 (0.92; 1.57)	1.08 (0.87; 1.33)	1.18 (0.87; 1.62)	-0.02 (-0.15; 0.11)

Hazard ratios for autism spectrum disorders, attention-deficit hyperactivity disorder and intellectual disability in the 1987-1996 birth cohort and mean difference in cognitive function score in the military conscription cohort comparing offspring born to mothers with HDP with those born to mothers without (= reference category) – within-sibship analyses by birth order of HDP occurence.
Model 1: adjusted for calendar year of birth only.
Model 2: adjusted for calendar year of birth, offspring sex, maternal age, parity, body mass index, smoking and pregestational diabetes. Maternal height, marital status and parental education and occupation in model 2 as these variables did not vary among full siblings within the same family.
Abbreviations: HDP = hypertensive disorders of pregnancy; HR = hazard ratio; CI = confidence interval.

© 2021 Brand JS et al. *JAMA Pediatrics*.

eTable 15. Associations Between Maternal Hypertensive Disorders of Pregnancy and Neurodevelopmental Outcomes in Offspring – Complete Case Analyses

	**Autism spectrum disorders**		**Attention-deficit hyperactivity disorder**		**Intellectual disability**		**Cognitive function score**	
	N total / N ASD	HR (95% CI)	N total / N ADHD	HR (95% CI)	N total / N ID	HR (95% CI)	N total	Mean difference (95% CI)
**MODEL 2**								
**Whole cohort**								
No HDP	553427 / 7913	REF	553427 / 18762	REF	553427 / 3741	REF	145836	REF
HDP	24118 / 478	1.27 (1.16; 1.40)	24118 / 882	1.07 (1.00; 1.15)	24118 / 229	1.33 (1.16; 1.52)	8204	-0.10 (-0.14;-0.06)
**Within-sibship**								
No HDP	228 / 94	REF	397 / 192	REF	122 / 59	REF	742	REF
HDP	207 / 108	1.42 (1.04; 1.94)	365 / 167	0.93 (0.73; 1.19)	111 / 50	1.07 (0.71; 1.62)	725	0.07 (-0.07; 0.20)

Hazard ratios for autism spectrum disorders, attention-deficit hyperactivity disorder and intellectual disability in the 1987-1996 birth cohort and mean difference in cognitive function score in the military conscription comparing offspring born to mothers with HDP with those born to mothers without (= reference category) – sensitivity analysis restricted to offspring with complete covariate data.
Model 2: adjusted for calendar year of birth, offspring sex, maternal age, parity, height, body mass index, smoking, pregestational diabetes, parental education, occupation and marital status.
The within-sibship models include offspring with at least 1 sibling and compare siblings discordant for maternal HDP, including all covariates listed in whole cohort analyses except for maternal height and marital status (model 2), as these variables did not vary among siblings within the same family.
N of within-sibship analyses refers to the number of offspring with siblings discordant on maternal HDP and each neurodevelopmental outcome.
Abbreviations: ASD = autism spectrum disorders; ADHD = attention-deficit hyperactivity disorder**;** ID = intellectual disability; HDP = hypertensive disorders of pregnancy; HR = hazard ratio; CI = confidence interval.

© 2021 Brand JS et al. *JAMA Pediatrics*.
